# Why Do High-Risk Patients Develop or Not Develop Coronary Artery Disease? Metabolic Insights from the CAPIRE Study

**DOI:** 10.3390/metabo12020123

**Published:** 2022-01-27

**Authors:** Martino Deidda, Antonio Noto, Christian Cadeddu Dessalvi, Daniele Andreini, Felicita Andreotti, Eleuterio Ferrannini, Roberto Latini, Aldo P. Maggioni, Marco Magnoni, Giuseppe Mercuro, on behalf of the CAPIRE Investigators

**Affiliations:** 1 Department of Medical Sciences and Public Health, University of Cagliari, 09042 Monserrato, Italy; martino.deidda@tiscali.it (M.D.); antonionoto@unica.it (A.N.); giuseppemercuro@gmail.com (G.M.); 2 Centro Cardiologico Monzino, IRCCS, 20138 Milan, Italy; daniele.andreini@cardiologicomonzino.it; 3 Department of Biomedical and Clinical Sciences “Luigi Sacco”, University of Milan, 20138 Milan, Italy; 4 Department of Cardiovascular Sciences, Fondazione Policlinico Universitario A. Gemelli IRCCS, 00168 Rome, Italy; felicita.andreotti@unicatt.it; 5 CNR Institute of Clinical Physiology, 56124 Pisa, Italy; ferranni@ifc.cnr.it; 6 Mario Negri Institute of Pharmacological Research—IRCCS, 20156 Milan, Italy; roberto.latini@marionegri.it; 7 ANMCO Research Center, Heart Care Foundation, 50121 Florence, Italy; maggioni@anmco.it; 8 Maria Cecilia Hospital, GVM Care&Research, 48033 Cotignola, Italy; 9 IRCCS Ospedale San Raffaele, Università Vita-Salute San Raffaele, 20132 Milan, Italy; magnoni.marco@tiscali.it

**Keywords:** coronary artery diseases, atherosclerosis, cardiovascular risk factors, metabolomics

## Abstract

Traditional cardiovascular (CV) risk factors (RFs) and coronary artery disease (CAD) do not always show a direct correlation. We investigated the metabolic differences in a cohort of patients with a high CV risk profile who developed, or did not develop, among those enrolled in the Coronary Atherosclerosis in Outlier Subjects: Protective and Novel Individual Risk Factors Evaluation (CAPIRE) study. We studied 112 subjects with a high CV risk profile, subdividing them according to the presence (CAD/High-RFs) or absence of CAD (No-CAD/High-RFs), assessed by computed tomography angiography. The metabolic differences between the two groups were identified by gas chromatography-mass spectrometry. Characteristic patterns and specific metabolites emerged for each of the two phenotypic groups: high concentrations of pyruvic acid, pipecolic acid, p-cresol, 3-aminoisobutyric acid, isoleucine, glyceric acid, lactic acid, sucrose, phosphoric acid, trimethylamine-N-oxide, 3-hydroxy-3-methylglutaric acid, erythritol, 3-hydroxybutyric acid, glucose, leucine, and glutamic acid; and low concentrations of cholesterol, hypoxanthine, glycerol-3-P, and cysteine in the CAD/High-RFs group vs the No-CAD/High-RFs group. Our results show the existence of different metabolic profiles between patients who develop CAD and those who do not, despite comparable high CV risk profiles. A specific cluster of metabolites, rather than a single marker, appears to be able to identify novel predisposing or protective mechanisms towards CAD beyond classic CVRFs.

## 1. Introduction

A risk factor (RF) is a specific condition, behavior or biological substrate statistically associated with a disease and shown to contribute to its pathogenesis, development or accelerated course. The Framingham Heart Study first investigated cardiovascular (CV) RFs, finding epidemiological relationships between cigarette smoking, blood pressure and cholesterol levels and the incidence of coronary artery disease (CAD) [1]. Nonetheless, seventy years later, CV disease (CVD) is still a major problem, causing disability and premature death around the world.

The latter consideration suggests that additional and unknown mechanisms may act independently of CVRFs or modulate the pathophysiological response to traditional CVRFs. Accordingly, despite the well known genetic influence on the development of CAD, as demonstrated by its high rate of inheritance (40–50%) [2,3], CAD cannot simply be considered as the expression of a transmitted predisposition, but, rather, as the result of multiple factors and genetic, epigenetic and environmental/behavioral interactions. 

The local microenvironment, resulting from the interplay between arterial mechanics, matrix remodeling, and lipid deposition, is able to modulate susceptibility to atherosclerosis development and progression. Furthermore, microenvironmental stimuli can affect other aspects of the microenvironment through collective adaptation. Our previous results have already shown that the metabolic profile of healthy coronary arteries is different from that of vessels affected by atherosclerosis, and that, in this setting, the metabolic fingerprint of microvascular dysfunction is distinguishable from that of stenotic disease, despite comparable cardiovascular risk factors [4].

The Coronary Atherosclerosis in Outlier Subjects: Protective and Novel Individual Risk Factors Evaluation (CAPIRE) study aimed to improve the knowledge of these interactions and, for this purpose, compared imaging and biochemical variables across four mutually exclusive phenotypes, defined by the presence/absence of CAD and RFs [5].

Metabolomics is a scientific tool recently applied to cardiology, which identifies and quantifies large numbers of metabolites in biological samples, providing immediate functional information. 

We previously investigated the functional-metabolic correlations of CAD, CVRFs, both or neither in a 2 × 2 phenotypic observational study [6]. Characteristic patterns and specific metabolic pathways were revealed in each phenotypic group of our patients, which included patients with or without CAD and with or without traditional CVRFs (ranging from CAD with high-risk profile to no CAD in the presence of multiple traditional CV RFs). In this study, we aimed to compare the metabolic fingerprint that characterizes and differentiates patients who, despite an equally high risk of CV, developed or did not develop CAD, thereby solving this unanswered medical question.

## 2. Results

No significant differences were found between the two groups in terms of anthropometric and clinical data (Table 1) or used drugs, except for acetylsalicylic acid (Table 2).

**Figure 1:**
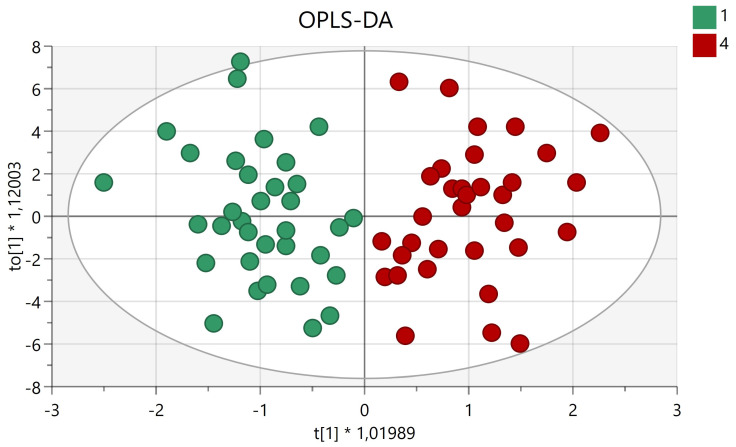
OPLS-DA score plot: the predictive (*x*-axis) and orthogonal (*y*-axis) components. Separation of classes is maximized along the predictive component, while the orthogonal component accounts for intra-class variability. Group 1 (green dots): no-CAD/high-RF vs. Group 4 (red dots): CAD/high-RF. The variable importance in projection (VIP) scores allowed further identification of the metabolites responsible for the separation between the two phenotypic groups.

We performed an OPLS-DA between CAD/high-RF subjects and no-CAD/high-RF subjects, observing a clear clustering that suggested the existence of different metabolic profiles and involved pathways (Figure 1). The R2 and Q2 values and the *p*-value of the corresponding analysis were: R2X: 0.417, R2Y: 0.744, Q2: 0.271, *p*-value: 0.013.

In detail, the CAD/high-RF group was characterized by high concentrations of pyruvic acid, pipecolic acid, p-cresol, 3-aminoisobutyric acid, isoleucine, glyceric acid, lactic acid, sucrose, phosphoric acid, trimethylamine-N-oxide, 3-hydroxy-3-methylglutaric acid, erythritol, 3-hydroxybutyric acid, glucose, leucine, and glutamic acid; and by low concentrations of cholesterol, hypoxanthine, glycerol-3-P, and cysteine compared to the no-CAD/high-RF group (Table 3).

## 3. Discussion

In this study, we focused on patients with high-risk CV profiles, with or without widespread CAD. The metabolomic analysis revealed specific metabolic fingerprints associated with each of the two phenotypes.

Some intermediates of the glycolysis pathways were found to be the major contributors to the cluster differences between the CAD/high-RF and no-CAD/high-RF groups, indicating a significant alteration of energy metabolism. Pyruvic acid, an intermediate in anaerobic glycolysis, appeared as the most significant metabolite in the comparison between the two patient groups. Lactic acid, which was in turn increased in CAD/high-RF patients, is a known marker of impaired energy metabolism in CAD [7], as a result of an impaired ability of the mitochondria to process pyruvic acid. Glycerol-3-phosphate, produced at the intersection of glucose and fat metabolism, the availability of which regulates energy and intermediate metabolism, exhibited a low serum concentration in the CAD/high-RF group.

Taken together, these findings denote a dysfunctional state of glucose oxidation, cellular redox and ATP production, gluconeogenesis, fatty acid esterification towards glycerolipids synthesis, and fatty acid oxidation [8].

Two other sugars, glucose and sucrose, were highly represented in the CAD/high-RF group. On the one hand, the direct and independent relationship between hyperglycemia and CVD, through the activation of multiple atherogenic mechanisms, is well known [9]. Furthermore, sucrose has been linked to the development of CAD, while data from the Coronary Artery Risk Development in Young Adults (CARDIA) study showed an inverse association between increased dietary sucrose intake and HDL cholesterol concentrations [10].

The ketone body 3-hydroxybutyrate, an important metabolite in ensuring ATP generation, was higher in the CAD/High-RF group. This molecule carries out cardioprotection in pathophysiological circumstances, primarily heart failure, providing an auxiliary fuel source and improving mitochondrial energetics [11]. Its increase in patients with CAD validates the hypothesis of their impaired energy metabolism.

Leucine and isoleucine, two branched-chain amino acids (BCAA), were highly represented when comparing CAD/high-RFs to no-CAD/high-RFs. The well known relationships between sugars and CAD mentioned above have implied the underestimation of the role of amino acids in the development and progression of CAD [12]. Conversely, a recent cohort study including incident CVD cases demonstrated a significant association of baseline leucine or isoleucine concentrations with higher CVD risk, after adjustment for potential confounders [13]. Furthermore, the results of a long-term (18.6 years of follow-up) prospective observational cohort of women, free of CVD at baseline, confirmed the positive association of total BCAA with CVD incidence [14].

Some studies, both in animal models and in humans, support our data, suggesting at least a partial explanation for the different evolution towards CAD of our two groups of subjects, with similarly high CV risk profiles, but different BCAA concentrations. The study by Li et al., using a mouse model of impaired BCAA catabolism, showed that BCAA accumulation selectively disrupts the utilization of mitochondrial pyruvate through the inhibition of pyruvate dehydrogenase complex (PDH) activity. This results in significant decreases in glucose uptake and oxidation, glycogen content and protein glycosylation, thus making the heart vulnerable to ischemia-reperfusion injury [15]. On the other hand, in a matched-pair case-control study Yang RY et al. demonstrated that BCAAs are significantly correlated with the development of CAD, independent of diabetes, hypertension, dyslipidemia, and body mass index [16].

Another amino acid that we found to be elevated in CAD/high-RF patients was pipecolic acid, a molecule originating from the lysine degradation pathway, possibly from intestinal bacterial enzyme metabolism. A recent study showed an association of lysine with CVD risk, but not with pipecolic acid [17]. Conversely, this molecule has been found to be elevated in diabetic corneas, suggesting a specific role in diabetes-induced diseases [18].

A further interesting result was the identification in the no-CAD/High-RF group of an elevated concentration of cysteine, a non-essential amino acid synthesized from methionine [19]. Cysteine is a powerful antioxidant capable of trapping reactive oxygen species (ROS) through sulfur residue, which determines disulfide bonds between two cysteine residues [19]. The increased presence of this amino acid in no-CAD/high-RF subjects can, therefore, be interpreted as a contrast to oxidative stress, in the presence of risk factors [20] and, ultimately, a form of protection against the development of CAD.

A recent accumulation of evidence suggests that alterations in the gut microbiome could play a role in CVD, with emphasis on heart failure and CAD [21]. In our study, CAD/high-RF patients exhibited high plasma concentrations of two gut microbiota-related metabolites, p-cresol and trimethylamine N-oxide (TMAO).

P-cresol is a methylphenol produced from tyrosine by the enteric pathogen, *Clostridium difficile*. In atherosclerosis-prone mice, p-cresol treatment activates macrophage micropinocytosis, leading to increased LDL uptake and higher hepatic/aortic fat deposits [22], prerequisites for a specific contribution to greater CV risk. P-cresol could also contribute to atherogenesis and thrombosis through the induction of ROS and cytotoxicity, and the production of inflammation/atherosclerosis-related modulators in endothelial and mononuclear cells [23].

TMAO is generated by gut microbes that, in anaerobic conditions, metabolize diet-derived molecules (choline, betaine, L-carnitine) to generate trimethylamine (TMA), which is converted to TMAO in the liver. Several studies have indicated a correlation between plasma TMAO levels and the risk of CAD. In particular, TMAO has been shown to (a) promote platelet reactivity; (b) potentially play a pro-thrombotic role; (c) cause vascular inflammation and activate inflammasomes; (d) worsen heart failure and chronic renal failure, causing a secondary increase in p-cresol; and (e) reduce the antiplatelet effect of acetylsalicylic acid [24,25,26,27]. In addition, prospective cohort studies have shown that increased plasma TMAO levels predict an elevated risk of major adverse CV events, such as myocardial infarction, stroke or death [28].

Unexpectedly, hypoxanthine and cholesterol, emblematic substances related to high CV risk, were less represented in the CAD/high-RF group. In our opinion, this apparent paradox can be explained by the more intensive control of CVRFs in the presence of CAD, as also suggested by the significantly higher intake of acetylsalicylic acid by these patients.

Overall, the results of this study demonstrate a distinct metabolic fingerprint in patients who develop CAD compared to those who remain free of CAD, despite a comparable CV risk profile. A cluster of specific metabolites, rather than the quantity of a single marker, seems to identify predisposing or, rather, protective mechanisms towards CAD, beyond classic CVRFs. These findings are in line with the hypothesis that the activity of “macro” CVRFs (hypertension, hypercholesterolemia, smoking, etc.) is modulated by micro-environmental (probably both genetically determined and acquired) factors able to address the development, or non-development, of atherosclerotic plaques.

The metabolic pathways that characterize each of the two groups, attributable to genetic heritage and/or to environmental/behavioral aspects, may contribute casually or be only the mirror of a complex interaction between traditional systemic CVRFs and molecular/local responses. In either case, specific metabolites appear to signal the attenuation, or even the prevention, of CAD (e.g., cysteine) or, conversely, the exacerbation or progression of the disease (e.g., BCAA, TMAO etc.), thus confirming the results of our preliminary study, carried out on coronary blood [4], that allowed us to distinguish healthy from stenosis-diseased or microvascular-impaired coronary arteries.

The current study is not intended to be exhaustive or conclusive but, rather, as a hypothesis-generating document capable of stimulating further research to elucidate individual susceptibility to CAD development. Data obtained from this kind (both clinical and “omic”) of investigation could help to improve our understanding of the natural history of atherosclerotic disease, but a complete understanding of the interplay between all the involved factors will require larger studies and the extensive use of integrated (clinical, metabolomic, genomic) approaches.

In this way, our results support GC-MS metabolomic analysis as a sensitive and specific tool for comparing different phenotypic groups and, in the future, for optimizing individual patient management and characterizing new therapeutic targets. 

## 4. Materials and Methods

### 4.1. Study Population

The study population was selected among both gender subjects aged 45 to 75 years, free of any previous ischemic heart disease, who underwent 64 slice (or superior) coronary computed tomography angiography (CCTA) in the outpatient clinics of the 11 centers involved in the study, because of suspected CAD. The main indications for CCTA were: (a) uninterpretable, equivocal, or contraindicated functional stress test and (b) new-onset chest pain syndrome at low–intermediate pre-test likelihood of CAD and (c) other indication including preoperative evaluation before valve or noncardiac surgery, elevated risk profile, arrhythmias, or atypical symptoms. [5]

From all the enrolled patients, on the basis of strict criteria of age (±5 years), sex (1:1 for the case and control groups), CVRFs and presence/absence of CAD, we selected 112 subjects. 

Coronary atherosclerotic plaque was defined as any recognizable structure of the coronary artery wall discriminated from surrounding pericardial tissue and epicardial fat; involvement of five coronary segments was selected as the cut-off to define diffuse CAD [5].

The definitions used to express the RFs were as follows:

- Family history: History of CAD in first-degree relatives, with onset <55 years for men and <65 years for women;
- Arterial hypertension: History of hypertension, current antihypertensive treatment, or recent blood pressure >140/90 mmHg;
- Hypercholesterolemia: Total cholesterol >200 mg/dL or <200 mg/dL with lipid-lowering medications
- Diabetes mellitus: Fasting plasma glucose >126 mg/dL, two-hour oral glucose tolerance test ≥200 mg/dL, isolated glycated hemoglobin ≥6.5%, or current use of insulin or oral hypoglycemic agents
- Smoking: Current or abstention <1 year.

According to the aforementioned criteria, all enrolled subjects were classified as high-RFs, given carriership of three or more RFs (>20% events at ten years), and assigned to two predefined groups:

- Subjects with CAD in >5/16 segments according to the American Heart Association classification and ³3 CVRFs (CAD/High-RFs; cases);
- Subjects without CAD but ³3 CVRFs (No-CAD/High-RFs; controls).

A peripheral venous blood sample was collected from all enrolled subjects after an overnight fast. The specimens, destined for metabolomic analysis, were processed and stored in a −70 °C freezer in a single dedicated biological bank. (SATURNE-1; Istituto di Ricerche Farmacologiche Mario Negri, Milano, Italia) [5].

The Ethics Committee of each participating center approved the study, which was performed according to the Helsinki Declaration. Written informed consent was obtained before inclusion.

Untargeted gas chromatography-mass spectrometry (GC-MS) analysis was then performed.

### 4.2. Preparation

A modified version of the procedure reported by Dunn et al. was followed [29]. Plasma samples were collected in EDTA-containing tubes and stored (−80 °C). A total of 400 μL of thawed plasma was transferred in Eppendorf tubes, treated with 1200 μL of cold methanol, vortex-mixed, and centrifuged for 15 min at 14,000 rpm (16.9 G). A total of 370 μL of supernatant was transferred into glass vials and evaporated to dryness overnight in an Eppendorf vacuum centrifuge. A total of 50 μL of a 0.24 M (20 mg/mL) solution of methoxylamine hydrochloride in pyridine was added to each vial; the samples were vortex-mixed and left to react for 17 h at room temperature. Next, 50 μL of *N*-Methyl-*N*-trimethylsilyltrifluoroacetamide was added and left to react for 1 h at room temperature. The derivatized samples were diluted with hexane (200 μL), with tetracosane (0.01 mg/mL) as the internal standard, just before GC-MS analysis.

For the GC-MS analysis, the samples were analyzed using an Agilent 5975C interfaced to the GC 7820 equipped with a DB-5ms column (J&W), at an injector temperature at 230 °C, a detector temperature at 280 °C, and a helium carrier gas flow rate of 1 mL/min. The GC oven temperature program was 90 °C initial temperature with a 1 min hold time and ramping at 10 °C/min to a final temperature of 270 °C with a 7 min hold time. A total of 1 μL of the derivatized sample was injected in split (1:5) mode. After a solvent delay of 3 min, mass spectra were acquired in full scan mode using 2.28 scans/s with a mass range of 50–700 Amu. Each acquired chromatogram was analyzed by means of the free software AMDIS (Automated Mass Spectral Deconvolution and Identification System; http://chemdata.nist.gov/mass-spc/amdis, accessed on 17 January 2021), which identified each peak through a comparison of the relative mass spectra and retention times with those stored in an in-house made library comprising 255 metabolites. Other metabolites were identified using NIST08 (the National Institute of Standards and Technology’s mass spectral database) and the Golm Metabolome Database (GMD, http://gmd.mpimp-golm.mpg.de/, accessed on 17 January 2021). Through this approach, 113 compounds were accurately identified, while 28 other metabolites were tentatively assigned, relying on GMD and NIST libraries. An AMDIS analysis produced an Excel data sheet that was successively subjected to chemometric analysis.

### 4.3. Statistical Analysis

An orthogonal partial least square-discriminant analysis (OPLS-DA) was conducted on SIMCA P+ 13 software (Umetrics, Umea, Sweden) and used to observe the data variance in a supervised mode. The data analysis was preceded by Pareto scaling, which reduced the relative importance of the large values, maintaining the overall data structure. The quality of the model was described by cumulative modelled variation in the X matrix R2X, cumulative modelled variation in the Y matrix R2Y, and cross-validated predictive ability Q2 values. To assess the significance of the model, a cross-validation analysis of variance (ANOVA) was applied. Discriminant metabolites, identified through variable importance in projection scores from the OPLS-DA, were considered specific for differences among phenotypic groups. In addition, a projection to latent structures regression (PLS) was conducted on SIMCA P+13 (Umea). A *p* value < 0.05 was considered statistically significant.

## Figures and Tables

**Table 1:** Demographic, anthropometric, and clinical data of the study population. Data are reported as mean ± SD for continuous variables and as numbers of affected/group sample.

	CAD/High-RF (Case, *N* = 56)	No-CAD/High-RF (Control, *N* = 56)
Age	61.8 ± 6.8	60.9 ± 7.5
M/F	34/22	34/22
Height (cm)	167.95 ± 8.75	167.61 ± 8.53
Weight (kg)	79.40 ± 15.08	76.98 ± 13.68
BMI (Kg/m^2^)	22.59 ± 5.51	21.95 ± 5.66
Abdominal circumference (cm)	100.04 ± 12.90	97.17 ± 11.60
CAD family history	34/56	34/56
Hypertension	52/56	53/56
Hypercholesterolemia	54/56	52/56
Diabetes mellitus	20/56	17/56
Tobacco	33/56	24/56
No CV-RFs	0/56	0/56

BMI: body mass index; CAD: coronary artery disease; CV-RFs: cardiovascular risk factors.

**Table 2:** Drugs use in the study population.

	CAD/High-RF (Case, *N* = 56)	No-CAD/High-RF (Control, *N* = 56)
No therapy	11/56	24/56 *
β-Blockers	10/56	16/56
ACE inhibitors	15/56	10/56
ARBs	8/56	13/56
CCB—dihydropyridines	5/51	3/53
CCB—no dihydropyridines	2/54	2/54
Diuretics	7/56	7/56
Potassium-sparing diuretics	0/56	0/56
Other antihypertensive drugs	0/56	0/56
Antiarrhythmic drugs	0/56	1/56
ASA	22/56	11/56 *
Clopidogrel	1/56	0/56
Statins	21/56	13/56
Other hypolipidemic drugs	1/56	5/56
Insulin	0/56	0/56
Other hypoglycemic drugs	5/56	7/56
Allopurinol	0/56	0/56

ACE: angiotensin-converting enzyme; ARBs: Angiotensin Receptor blockers; CCB: calcium channel blockers; ASA: acetyl salicylic acid. * *p* < 0.05 vs. CAD/High-RF.

**Table 3:** List of the most significant metabolites, obtained by multivariate statistical analysis, discriminating patients with CAD/high-RFs from no-CAD/high-RF subjects.

Metabolite	Trend in CAD/High-RFs	VIP Value
Pyruvic acid	↑	3.09
Pipecolic acid	↑	2.44
p-Cresol	↑	2.37
3-Aminoisobutyric acid	↑	2.28
Isoleucine	↑	2.18
Cholesterol	↓	1.96
Lactic acid	↑	1.64
Sucrose	↑	1.63
Hypoxanthine	↓	1.51
Phosphoric acid	↑	1.39
Trimethylamine-N-oxide	↑	1.27
3-hydroxy-3-methylglutaric acid	↑	1.14
Erythritol	↑	1.12
3-hydroxybutyric acid	↑	1.09
Glycerol-3-P	↓	1.08
Glucose	↑	1.03
Leucine	↑	1.01
Cysteine	↓	1.00
Glutamic acid	↑	1.00

## Data Availability

Data supporting the findings of this study are available upon reasonable request to CAPIRE Steering Committee.

## Appendix A

Steering Committee

A. Maseri † (Chairman; Firenze), D. Andreini (Milano), S. Berti (Massa), M. Canestrari (Fano), G. Casolo (Lido di Camaiore), D. Gabrielli (Roma), R. Latini (Milano), M. Magnoni (Milano), P. Marraccini (Pisa), T. Moccetti (Lugano), M.G. Modena (Modena)

Coordinating Center: A.P. Maggioni, M. Gorini, F. Bianchini, I. Cangioli, A. Lorimer (Centro Studi ANMCO Firenze)

Imaging Core Laboratory: D. Andreini, G. Pontone, E. Conte (Centro Cardiologico Monzino Milano)

Centralized biobank and biomarker core laboratory: D. Novelli, F. Gaspari, S. Ferrari, A. Cannata, N. Stucchi, M. Fois, R. Bernasconi, G. Balconi (Istituto Mario Negri, Milano and Bergamo), T. Vago, T. Letizia (Ospedale Luigi Sacco, Milano), B. Bottazzi, R. Leone (Istituto Clinico Humanitas, Rozzano).

Central ECG Reading: I. Suliman (Centro Studi ANMCO, Firenze)

Psychologists CRF Group: M. Sommaruga † (IRCCS Salvatore Maugeri Unità di Psicologia, Milano), P. Gremigni (Dipartimento di Psicologia Università di Bologna)

Participating Centers and Investigators

Fano, Ospedale S Croce (R. Olivieri); Fermo, Ospedale Civile A. Murri (L. Pennacchietti); Lido di Camaiore, Nuovo Ospedale Versilia (M. Magnacca); Lugano, Cardiocentro Ticino (M.G. Rossi, E. Pasotti, T. Moccetti); Massa, FTGM - Stabilimento di Massa (A. Clemente); Milano, Centro Cardiologico Monzino (D. Andreini, G. Pontone, S. Mushtaq); Modena, Ospedale Policlinico (E. Mauro, G. Boriani); Parma, AOU. di Parma (F. Pigazzani); Pisa, AOU Pisana (L. Faggioni); Pisa, FTGM—Stabilimento di Pisa (M. Ciardetti); Udine, AOU SM della Misericordia (M. Puppato)
